# Advances in understanding the formation and fate of B-cell memory in response to immunization or infection

**DOI:** 10.1093/oxfimm/iqab018

**Published:** 2021-09-11

**Authors:** Liam Kealy, Kim L Good-Jacobson

**Affiliations:** 1 Department of Biochemistry and Molecular Biology, Monash University, Clayton, Victoria, Australia; 2 Infection and Immunity Program, Biomedicine Discovery Institute, Monash University, Clayton, Victoria, Australia

**Keywords:** memory B cells, germinal center, antibody, SARS-CoV-2

## Abstract

Immunological memory has the potential to provide lifelong protection against recurrent infections. As such, it has been crucial to the success of vaccines. Yet, the recent pandemic has illuminated key gaps in our knowledge related to the factors influencing effective memory formation and the inability to predict the longevity of immune protection. In recent decades, researchers have acquired a number of novel and powerful tools with which to study the factors underpinning humoral memory. These tools have been used to study the B-cell fate decisions that occur within the germinal centre (GC), a site where responding B cells undergo affinity maturation and are one of the major routes for memory B cell and high-affinity long-lived plasma cell formation. The advent of single-cell sequencing technology has provided an enhanced resolution for studying fate decisions within the GC and cutting-edge techniques have enabled researchers to model this reaction with more accuracy both *in vitro* and *in silico*. Moreover, modern approaches to studying memory B cells have allowed us to gain a better appreciation for the heterogeneity and adaptability of this vital class of B cells. Together, these studies have facilitated important breakthroughs in our understanding of how these systems operate to ensure a successful immune response. In this review, we describe recent advances in the field of GC and memory B-cell biology in order to provide insight into how humoral memory is formed, as well as the potential for generating lasting immunity to novel pathogens such as severe acute respiratory syndrome coronavirus 2.

## INTRODUCTION

Humans are constantly coming into contact with pathogens that pose a significant threat to their health. Given the prevalence, variability and potential risk of these pathogens, we are reliant on our immune system to safeguard us from possible catastrophe. While the majority of pathogens can be successfully neutralized by our immune defences, there is a minority that, without intervention, can result in death of the host. One critical example of such a pathogen is severe acute respiratory syndrome coronavirus 2 (SARS-CoV-2), the novel coronavirus responsible for causing Coronavirus Disease 2019 (COVID-19). This disease was first presented in patients in Wuhan, China at the end of 2019 and was officially declared a pandemic by the World Health Organization on 11 March 2020 [1]. With 196 million cases confirmed worldwide and 4.2 million lives lost as of July 2021, COVID-19 has fast become the most challenging pandemic of this century [2].

In order to mitigate the spread of the virus, strict public health measures were implemented around the world with varying degrees of success. While these measures have been largely effective at saving lives, they have come at a significant cost to the global economy and public well-being. In order to find a sustainable solution to these issues and a resolution to this pandemic, the development and distribution of a safe and effective SARS-CoV-2 vaccine quickly rose to the top of the agenda for leaders, scientists and policymakers in 2020. Consequently, as of July 2021, there are now 77 vaccine candidates in preclinical development, 98 in clinical trials on humans, 32 that have reached the final stages of testing, 5 that have been approved for emergency use and 11 that have been fully approved for use in several countries by their respective health regulatory agencies [3].

For a vaccine or pathogen to successfully generate lasting immunity, they must elicit B-cells to form long-lived antibody-secreting plasma cells and memory cells, mainly via the germinal centre (GC) reaction [4]. GCs are microanatomical structures located within lymphoid organs that form during an immune response. Within GCs, activated B-cells undergo proliferation and diversification of their immunoglobulin genes in order to raise the affinity of their respective antibody. Long-lived plasma cells offer protection up to the remainder of the host’s lifetime via the production of tailored antibodies [5]. These antibodies are multifunctional: they can neutralize the activity of their target, regulate the efficacy of innate immune cells, and activate the complement pathway, to help disable and/or destroy the pathogen of interest [6]. One of the fundamental features of a successful adaptive immune response is its ability to deliver a superior response upon re-infection with the same pathogen. This is achieved by the memory B-cell and T-cell populations that are produced by the primary immune response. These cells are capable of persisting in a quiescent state for decades before they are re-exposed to their cognate antigen [7] and possess the ability to generate potent and accelerated responses upon re-stimulation [8].

The mechanics underlying the formation, function and heterogeneity of the memory B-cell subset have in the past been elusive. Technological and methodological advances over recent years, however, have provided new opportunities to gain a better understanding about the different types of memory B-cells that can arise under different pathogenic conditions as well as of how they function. Similarly, novel approaches to studying activated B-cells and the GC reaction have led to a better understanding of the factors which give rise to different types of memory B-cells, as well as of the factors which underly the fate decision to become a memory B-cell or a plasma cell. Cutting-edge approaches to studying the GC under single-cell resolution have the potential of unveiling more novel insights into the molecular regulation of B-cell fate decisions, which in turn may assist in the development of vaccines and/or therapies for pathogens that are newly emerging or pose a continuing challenge to human health.

COVID-19 and the roll-out of vaccines have put a spotlight on some of the many long-standing questions about our ability to form lasting immunity. While many of these important questions apply to T cells, this review will focus on those related to B cells and antibody formation. For instance, what are the key biological and molecular factors which underpin immune memory formation and persistence? Can these factors be manipulated in order to ameliorate the quality and durability of the response? Do differences in the humoral immune response generated by different individuals reflect differences in patient morbidity? Finally, to what degree might we be able to predict the quality and longevity of humoral memory during the early stages of an immune response to a pathogen or vaccine? In order to provide some insight into these questions, we dissect recent technical advances and breakthroughs in the understanding of GC and memory B-cell biology, which have enabled researchers to better understand the generation and persistence of long-term humoral memory to challenging pathogens such as SARS-CoV-2.

## DEFINING MEMORY B-CELLS IN DIFFERENT IMMUNOGENIC CONTEXTS

Three major questions have underpinned research into memory B cells and their ability to contribute to immune protection: (i) How can we phenotypically define memory B cells? (ii) How does the memory B-cell population provide an enhanced response upon recall? (iii) Are there deterministic factors that specifically initiate the differentiation of B cells into memory? Memory B cells have been notoriously difficult to study ex vivo, compared to other humoral system subsets such as GC B cells or plasma cells. Low frequencies of antigen-specific memory B cells make it difficult to study the behaviour of these cells in tissues. Numerous studies have been performed to date using a combination of markers to identify murine memory B cells, however, the lack of a single defining memory B-cell marker or transcription factor precludes conditional deletion of genes solely within the memory B-cell subset, a method that would underpin a powerful new way to investigate memory B-cell formation and function. Despite these limitations, development of important tools, such as antigen tetramers, deep-sequencing and B-cell receptor (BCR) repertoire analysis, have enhanced our understanding of the factors that affect memory B-cell fate and have additionally provided us with an increased appreciation for the heterogeneity that exists within the memory B-cell subset (recently reviewed in Ref. [9]). In particular, research findings over the last few decades have linked phenotypic and BCR affinity heterogeneity of these cells with differential roles in formation and function. Adding an extra layer of complexity, advances in techniques have distinguished new memory B-cells subsets in humans, mice, tissues and in response to different immunogens.

### Assessing the heterogeneity of human B-cell memory subsets

Understanding defining features of the memory B-cell population in healthy cohorts enables isolation for functional and molecular characterization. It also facilitates the identification of unique disease-induced changes in phenotype and function of B cells in patients, as well as the potential for these changes to be used as clinical biomarkers of a dysregulated functional process within tissues. In humans, memory B cells are often defined by the marker CD27 [10, 11], and this definition has been further developed over the year to segregate out subsets based on isotypes and other markers (reviewed in Refs [12, 13]). Evolving methodologies, such as an increase in the molecules able to be assessed by flow cytometric immune profiling or by mass cytometry, has illuminated the heterogeneity that exists within and across tissues such as bone marrow, tonsil, lymph nodes, spleen, gut and peripheral blood [14, 15]. Glass *et al*. recently performed a mass cytometry screen for hundreds of surface molecules expressed by B cells [15]. In this way, the researchers behind this study were able to identify and catalogue surface markers that were unique to specific classes of memory B cells, such as CD45RB, a marker also noted by Weisel *et al*. [14]. Both studies also identified distinct CD27^−^ memory B-cell subsets in multiple tissues [14, 15]. These studies have provided a comprehensive guide to shared and unique markers of memory B-cell subsets across tissues in humans, an important step for understanding how the memory B-cell population is effective at providing tailored protection against pathogens. This will be advanced by future studies investigating whether tissue-specific signals promote particular subsets, and whether phenotypic subsets are linked to unique functions in different tissues of the body.

### Novel tools to study the factors underpinning B-cell memory

Historically, a key approach to study memory B cells *in vivo* is via immunizing mice with a haptenated protein [e.g. the hapten (4-hydroxy-3-nitrophenyl)acetyl conjugated to a protein such as keyhole limpet haemocyanin], the response to which has been extremely well-characterized with the established tools needed to investigate antigen-specific memory B cells [16–19]. In recent years though, attention has turned to studying infection-induced memory B-cells *in vivo*. Due to the rarity of antigen-specific memory B cells, antigen tetramers were developed that can identify these cells during a polyclonal response to infection [20]. The implementation of these tools, in addition to the recent advances in single-cell and phylogenetic analysis of B-cell clones, has had a significant impact by allowing researchers to map the fate and behaviour of antigen-specific memory B cells under different pathogenic conditions, in both humans and mice.

Upon secondary exposure to antigen, memory B cells can immediately differentiate into plasma cells that possess a higher affinity and faster rate of antibody production than those produced during the primary response. Alternatively, they may re-enter the GC to further revise their BCR in a context-specific manner, although to what extent this occurs has been a source of recent contention [21–24]. Importantly, the use of B-cell tetramers has enabled dissection of pathogen-specific behaviour of memory B-cell subsets. For example, Pepper and colleagues investigated the role of antigen-specific memory B-cell subsets produced in response to *Plasmodium* infection [25]. Discordant views of the function of IgM^+^ memory B cells had arisen in previous years from studies of humans [12] and murine models [17, 26, 27], particularly whether they participated in producing plasmablasts during secondary responses. Through analysing tetramer-binding B cells in a mouse model of malaria, the authors demonstrated that even when in competition with IgG^+^ memory B cells, IgM^+^ memory B cells generate robust and rapid plasmablast responses upon re-infection with malaria; challenging the view that IgM^+^ memory B cells represent a less-impactful memory population [25]. Further, while a number of studies with model antigens have demonstrated the recruitment of a subset of memory B cell into secondary GCs [17, 26], one recent study observed that memory B cells generated in response to Flaviviral infections and vaccinations did not undergo further affinity maturation in recall responses [28]. Concordant findings have now been made using model antigen and influenza infection models. Utilizing photoactivable ‘confetti’ mice (in which stochastic recombination of fluorescent alleles allows tracking of B-cell clones and their progeny), parabiosis and/or single-cell BCR repertoire analysis has also determined that GC re-entry occurs only in a minority of memory B cells [24, 29]. Thus, the generation of novel tools, such as those described above, is of critical importance in understanding the heterogeneity and function of memory B-cell subsets to infection.

### Understanding the generation of humoral memory during chronic infection

During the immune response to chronic or recurrent infectious diseases, for which disease-acquired immunity is not easily established (such as _Err_RptHIV, tuberculosis and malaria), fundamental changes to the memory B-cell compartment can occur which result in a diminished ability by these cells to respond effectively to stimuli *in vitro* [30, 31]. The most prominently observed change is an increase in a distinct memory B-cell subset, not normally detected in healthy donor peripheral blood, originally termed ‘atypical’ (generally defined as CD20^+^CD27^−^CD10^−^CD21^lo^). It remains controversial whether the detection of these cells is a consequence of a pathogen-specific mechanism that generates a new class of defective memory B cells, or due to the recruitment and/or redistribution of normally tissue-resident memory B cells to a different anatomical site via peripheral blood. As this subset has been recently reviewed [32, 33], we will not do so in detail here, except to observe that while this subset has been linked to dysfunction in immune memory, a number of recent studies have suggested that it may be a normal component of the B-cell response to infection [32, 34, 35]. For example, using recombinant Haemagglutinin probes or B-cell tetramers to analyse antigen-specific B-cells post either influenza infection, *Plasmodium* infection or vaccination, as well as single-cell RNA-sequencing to analyse gene expression, Sutton *et al*. demonstrated that cells of the ‘atypical’ phenotype emerged in healthy donors [34]. In line with these studies, the recent advanced cytometric analyses of B-cell heterogeneity described in ‘Assessing the heterogeneity of human B-cell memory subsets’ section identified subsets of a similar phenotype in assessed tissues [14, 15]. Which signals drive the recruitment of this cell subset into the peripheral blood during infection, and whether it is a useful biomarker of functional changes in the tissue, is an area of important ongoing research. On a broader note, it is still unclear how chronic infection disrupts the effectiveness of B-cell memory, thereby limiting our toolkit to therapeutically intervene to enhance immune protection.

## DIFFERENT TYPES OF B-CELL RESPONSES LEAD TO A HETEROGENOUS MEMORY B-CELL POPULATION

Adaptive immune cells are able to respond, through unique antigen receptors, to a near limitless array of potential foreign pathogens. Upon activation by antigen, B cells can proceed down an array of different differentiation pathways which differ depending on a variety of factors, such as the type, timing and anatomical location of the response. The culmination of all of these different potential pathways is a heterogenous constellation of B cell progeny that, together, are able to provide lasting protection against the antigen that created them through the production of highly specific, tailored antibodies and an adaptable fleet of memory B cells.

### T-independent memory B cells

B cells possess the ability to respond to different types of antigen in a number of different ways. The different types of antigens which activate naive B cells can be divided into two main categories: T-dependent (TD) and T-independent (TI). TD antigens describe a broad array of primary proteins, or haptens conjugated to proteins, which rely on MHC-dependent signals from CD4^+^ T cells to deliver a long-lived, high-affinity antibody response [36]. Meanwhile, TI antigens contain repetitive epitopes, or molecules that can be recognized by pattern recognition receptors, which enable B cells to bypass the need for T-cell help [37]. As a result, B cells can respond to TI antigens quickly, albeit with lower affinity and a faster resolution than is seen following TD activation [38]. It was long believed that TI responses do not elicit B-cell memory in any form. However, multiple studies performed over the last two decades identified a different, yet very present class of TI memory B cells. NP-Ficoll immunization induced detectable antigen-specific memory B cells that persisted months post-immunization [39]. B1b-derived memory B cells possessed the capacity to produce boosted responses upon secondary exposure, in the absence of antigen-specific IgG [40]. Furthermore, long-lived plasma cells are generated following TI immunization and these cells migrate to the bone marrow in order to provide the host with lasting antibody protection [41]. The findings described above are important for changing the dogma that TI antigens do not promote long-term immune memory, however, the memory cells which are created by this type of stimulation are intrinsically different from those produced by a classical TD response. For this reason, it is unclear whether TI-derived memory B cells would produce the enhanced response required for effective, long-lived immune protection.

### Formation of TD memory B cells

TD responses drive the formation of affinity-matured memory B cells that are intrinsically wired for enhanced responsiveness [8, 16, 42]. B-cell fate decisions are driven via the integration of specific signals from the microenvironment as they move through the tissue. The movement of activated B cells is choreographed by chronologically coordinating chemokine receptor expression on activated B cells so as to respond to the complex milieu of chemokine gradients which delineate the various regions of lymphoid tissue. B-cell follicles are defined by high concentrations of CXCL13, which is recognized by CXCR5 on B cells [43]. Inducing CXCL13 expression in peripheral tissues such as the lungs in response to interferon signalling has been found to be both necessary to support ectopic GC formation via recruitment of CXCR5^+^ B cells [44]. Following TD antigen recognition, B cells will interact with CD4^+^ T cells, at the border between their respective zones, to receive CD40 ligand (CD40L) and cytokine signalling before homing back into the B-cell follicle [36]. Activated B cells can differentiate into memory B cells in a GC-independent manner throughout the response [45, 46]. In the GC B cell differentiation pathway, B cells receive IL-21 support from T follicular helper cells (T_FH_) to underpin the formation of a fully functional GC [47, 48]. GCs operate as sites of affinity maturation through clonal expansion, somatic hypermutation (which alters the specificity of the BCR introducing point mutations into the V region of the immunoglobulin gene [4]) and selection of reactive B cells to differentiate into long-lived plasma cells and memory B cells. Modulation of B-cell quality via affinity maturation within the GC is central to protective humoral responses. For this reason, the GC represents a key system underpinning the formation of the memory and plasma cell repertoire.

### Tailoring of memory B-cell subsets by cytokines

Cytokines help to establish the B-cell response as well as drive the tailoring of the eventual antibody to match the type of infection, resulting in an additional layer of heterogeneity to the plasma cells and memory B cells generated by the immune response. Th2-associated cytokines such as IL-4, IL-5, IL-6 and Type 1 interferons have all been found to play important roles in promoting B-cell activation and in directing B cells towards different isotype classes [49, 50]. Interferon-gamma tailors B cell responses to viral infection and is typically associated with Th1-cells [51]. In this way, different classes of memory B-cells can be generated using specific combinations of cytokines. As in the case of the CD4^+^ memory T cells [52], these different classes of memory B cells may differ in their functionality, location and effectiveness at dealing with a particular type of pathogen. B cells will respond to Th1-cell-biased microenvironment by expressing the transcription factor T-bet, which is retained in a subset of the resulting memory B cells [53]. While T-bet is known to drive IgG2 class-switching, it has other important roles including plasma cell development and tissue homing [54–57]. While T-bet and Eomes play opposing roles in Th subsets, Eomes does not have the converse function in B cells, as it does not regulate the B-cell behaviour during either a Th1 or Th2-cell biased response [58].

### Tissue-specific functions of memory B-cell subsets

In addition to the type of stimuli, the anatomical location of memory B cells has also recently been shown to play a critical role in determining the behaviour and function of memory B cells. A number of putative tissue-specific or tissue-resident memory B cells have been identified in humans [14, 15, 59, 60] and mice [60–64]. These phenotypically and functionally distinct memory B cells play unique and critical roles at various different locations within the body including the lungs, tonsil, gut and vagina [14, 57, 59, 61]. Furthermore, a recent study of human peripheral blood and splenic memory B-cell repertoires in individuals of different age groups demonstrated that in addition to a circulating subset, there appeared to be ‘archiving’ of memory B-cell clones in the splenic marginal zone over time [65]. Investigating unique changes in tissue-specific memory B cells, in addition to defining the residency of these subsets (as discussed below), provides further evidence of the importance of the functional diversity within the memory B-cell repertoire.

A number of questions arise from the identification of memory B-cell subsets in the tissue. Can they be formed from a local GC response, or do they migrate into the tissue, such as those recruited into the vagina in response to genital herpes infection [57]? Once in the tissue, do they establish residence, or can they continue to migrate into the peripheral blood to be recruited into other tissue sites? Recently, a number of research groups dissected the formation, persistence and location of memory B cells formed to influenza infection in the lung, spleen and lymph nodes [60–62]. While CD69-expressing memory B cells had previously been described persisting in the lung [64], Randall and colleagues utilized tetramers and parabiotic mice to demonstrate that antigen-specific memory B cells in the lung did not recirculate [61]. Comparison of memory B cells with different T-bet expression levels, in a study also utilizing parabiotic mice, revealed that T-bet expression segregated populations, describing different classes of memory B cells with distinct tissue residency, migration and functional properties [60]. T-bet^hi^ memory B cells preferentially resided within the spleen, while T-bet^lo^ memory B cells entered circulation [60]. Tissue-resident memory B cells have the potential to be useful therapeutic targets, such as to enhance vaccine effectiveness, but this is still a nascent area of research. The potential for targeting will be facilitated by future studies designed to understand distinct signatures and functional capabilities of tissue-specific memory B cells in humans. It will also increase our understanding of whether differences detected in peripheral blood B cells are due to the recruitment of tissue-specific subsets and/or indicative of dysregulation.

## STUDYING THE GC UNDER A NEW RESOLUTION TO DECIPHER B-CELL FATE DECISIONS

An exciting area of research has focused on testing whether the GC reaction can be therapeutically manipulated in order to enhance or repair immune memory and antibody formation, such as for treating HIV [66, 67]. One key hurdle that researchers are tackling is identification of factors regulating the decision point at which B cells exit the GC to form the memory population. Endeavours to shed light on the inner regulatory workings of the GC up until now have been limited by the research tools at our disposal. The cyclic properties of the GC, along with the absence of a unique single marker for each of the different zones of the GC, precludes classical conditional deletion studies from elucidating some of the major unknowns regarding the GC to memory B-cell transition. In addition, historically most transcriptomic approaches have required the pooling and lysing of large numbers of GC B cells. While informative, these approaches do not account for the heterogeneity that exists within the GC or for the fact that regulation of B-cell fate is fundamentally conducted at a single-cell level. For these reasons, different approaches had to be taken in order to gain better insight into the inner GC dynamics. Single-cell resolution is vital in order to accurately study the intercellular dynamics and regulatory factors that determine cell fate decisions within the GC. As a result of the recent implementation of novel tools such as single-cell sequencing, B-cell biologists have begun to shed new light on some of long-standing questions in the field pertaining to GC B-cell fate.

### Regulating the fate of B cells within the GC

Through single-cell technology, we have gained a novel opportunity to study the differences between cells from the same GC [24, 29, 68, 69], between GCs [70] and observe, in pseudo-time, the commitment and transition of GC B cells into either plasma cell or memory B-cell precursors and have acquired a new way with which to more closely interrogate the precise intrinsic factors that determine GC B-cell fate [69].

One recent landmark discovery which also utilized single-cell sequencing to interrogate GC dynamics was reported by Kennedy *et al.* after they uncovered a third zone within the GC—termed the ‘grey zone’ or ‘proliferating dark zone’. Using an assortment of sequencing strategies involving bulk and single-cell transcriptomic, proteomic and epigenomic analyses a distinct GC zone with a unique transcriptional signature was identified that had previously been formally characterized as belonging to the dark zone. It was found that this new zone is dedicated to clonal expansion, while the remaining section of what was previously the dark zone is dedicated to differentiation prior to LZ re-entry [68]. This finding prompts re-evaluation of our presumptions regarding the spatiotemporal organization of the GC. Future studies may be able to use this extensive data set to investigate whether there is a correlation between particular epigenetic and transcriptional changes within zones and a propensity to adopt a memory B cell or plasma cell precursor fate. Identification of memory B cell and plasma cell precursors within the GC has been a hot topic of research, and recent studies have also utilized single-cell sequencing to identify gene signatures for precursors within the GC [69]. Taken together with the Kennedy data set [68] as well as single-cell sequencing of influenza-specific GC and memory B cells from different tissues [62], the identification of these precursor signatures is highly impactful since they enable the field to predict key factors that may dictate GC B-cell fate upon exit [69].

### Molecular and transcriptional factors which dictate GC B-cell fate

After decades of research into the molecules which drive B-cell activation, we now have remarkable insight into a plethora of intrinsic factors, such as transcription factors [71] and epigenetic regulators [16, 72–78], that orchestrate B-cell fate during an immune response. Transcription factors are key regulators of differentiation of GC B cells into plasma cells [71]. After sufficient time cycling between the different zones of the GC, B-cell clones will eventually depart the GC as a plasma cell or as a memory B cell. Plasma cell differentiation begins within the GC and is accompanied by the expression of *Prdm1*, which encodes for the key transcription factor, BLIMP-1 [79]. BLIMP-1 is responsible for repressing transcription factors associated with B-cell identity, such as PAX5, and upregulating the expression of genes associated with antibody secretion and plasma cell identity [80, 81]. A recent study demonstrated that low levels of *Prdm1* expression begin in the proliferative centroblasts of the DZ, even in the absence of T_FH_ cells, and that these cells, while not fully committed to becoming plasma cells, did appear to be primed for this fate [82]. In addition to BLIMP-1, the transcription factors IRF4 and XBP1 have been shown to play critical roles in regulating the plasma cell transcriptional program. Conditional deletion of IRF4 resulted in the inability of B cells to class-switch and GC-derived and memory B-cell-derived plasma cell differentiation was defective [83]. Downstream of BLIMP-1, XBP1 regulates the intracellular secretory apparatus and raises the capacity for protein synthesis in order to prepare the plasma cell for vigorous antibody secretion [84]. An important feature of long-lived plasma cells is their migration from secondary lymphoid organs to the bone marrow to take up long-term residence; this too is mediated by a transcription factor, c-Myb, which regulates the responsiveness of plasma cells to the chemokine CXCL12 [85] and thus their migration out of the secondary lymphoid organs to the bone marrow.

In contrast to GC B cells and plasma cells, memory B cells have very similar transcriptional profiles to naïve B cells, which has made identifying potential transcription factors regulating memory B-cell differentiation difficult [8, 86–88]. The transcription factor BOB.1 is one transcription factor that has been correlated with modulation of the memory B cell versus plasma cell fate outcome. *In vitro* experiments, in which BOB.1 was either overexpressed or knocked down in B cells, demonstrated that high levels of BOB.1 favour upregulation of memory B-cell markers, while low levels predispose B cells to become plasma cells [89]. Next steps for understanding the role of BOB.1 would include determining whether these findings are replicated in GC responses *in vivo.* Several transcription factors have been reported to regulate the GC to memory B-cell transition, such as BACH2, HHEX (in concert with cofactor Tle3), SKI and KLF2 [90, 91]. The conditional deletion of *Hhex* resulted in decreased memory B-cell output from the GC and a decrease in numerous genes associated with memory B cells; crossing of *Hhex*-deficient mice to a Bcl2 transgenic restored memory B cell to GC ratio to that equivalent of mice heterozygotes for the floxed allele [90]. Overexpression of HHEX, Tle3, SKI or KLF2 all promoted 2- to 4-fold changes in the ratio of GC to memory B cells, mainly due to a larger reduction in the frequency of GC B cells transduced with the candidate molecule compared to the empty vector control, than the reduction observed between candidate molecule and control-transduced memory B cells [90]. This is concordant with a previous study that demonstrated increased proliferation of B cells upon overexpression of KLF2 or SKI [92]. BACH2 is also more highly expressed in memory B cell precursors within the GC compared to other GC subsets [93]. While BACH2 regulates GC-derived memory B cell differentiation, it also plays an important and well-established role within the GC, indicating that factors other than BACH2 must also be playing a key role in regulating the GC to memory B cell transition [93–95].

Aside from transcription factors, a number of other molecules along with positioning within the GC have also emerged as a key indicator of precursor memory B cells. CCR6 and Ephrin-B1 are both extracellular receptors that serve as markers for memory B-cell precursors within the GC within the light zone [96, 97]. GC B cells expressing either of these molecules were transcriptionally and phenotypically associated with a memory B-cell phenotype, however, the functional mechanisms of how or why these molecules are expressed on these precursors are still unclear. Further, there is little known about the upstream extrinsic factors that induce these molecules and begin the differentiation process. Within the GC, recent findings have also shown that metabolic reprogramming, via an axis of decreased CD40 signalling strength and mTOR1 levels, along with increased Bcl2, is another important factor in regulating memory B-cell exit and survival [93]. IL-9 signalling by T_FH_ cells has also been shown to be playing a role in memory B cell differentiation out of the GC, however, it is not yet clear whether IL-9-mediated induction of memory B-cell fate is a generalizable feature across different infection models [9, 98, 99].

**Figure 1:**
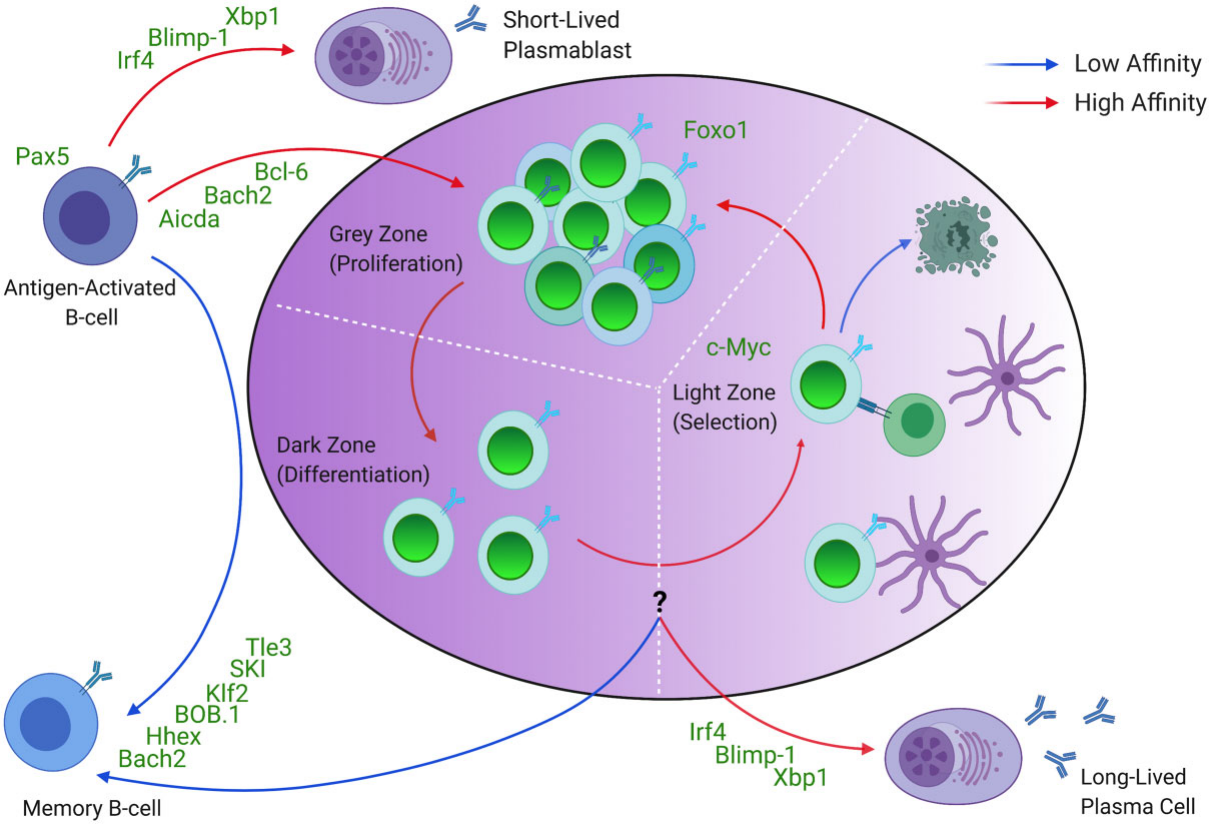
Schematic demonstrating how affinity and transcription factors regulate fate decisions within the different zones of the GC. The role of affinity, location and other molecules is driving memory B cell differentiation over a plasma cell fate has not yet been fully elucidated.

These studies demonstrate that alteration of expression of numerous molecules promotes a memory B-cell fate over either a GC or plasma cell fate (Fig. 1). However, more work needs to be done to untangle the differential roles of these molecules in GC B cells versus specifically promoting memory B-cell differentiation by, e.g. testing in a TI model. However, two recent studies have given insight into the generation of GC-independent memory. BAFF receptor expression on B cells was found to be playing a key role in mediating the commitment of activated B cells into the early memory B-cell fate [100, 101]. While BAFF-R was dispensable for GC-derived memory B-cell differentiation, GC-independent memory B cells were diminished in the absence of BAFF-R, and reciprocally were expanded when BAFF signalling was enhanced [100, 101]. In this way, BAFFR appears to be playing a context-specific role in mediating memory B-cell differentiation. An improved understanding of the mechanistic steps that lead to key fate decisions will be required before new therapeutic avenues to elicit memory B-cell formation or fine-tune their activity can be pursued.

### Role of the BCR in dictating B-cell fate within the GC

Recent research has aimed to determine whether BCR affinity has a causative or correlative role in determining the fate of B cells within the GC. It is well established that both antigen engagement and T_FH_–B cell signalling are critical in regulating GC B-cell fate [93, 102–105]. Studies comparing BCR affinity between memory B cells and plasma cells appear to point to plasma cells as possessing higher affinity than their memory B-cell counterparts (Fig. 1), and experimental augmentation of centrocyte–T_FH_ interactions leads to an increase in plasma cell differentiation, indicating these signals are important for plasma cell selection [102, 106, 107]. Lower-affinity GC clones possessed higher *Bach2* expression and thus correlated to an increased potential to differentiate into a memory B cell [106]. BrdU pulse-labelling studies demonstrated that memory B-cell output precedes plasma cell emigration in an adoptive transfer immunization model [108]. To explain this observation, it was proposed that GC B-cell departure as either a memory B cell or a long-lived plasma cell is regulated by a ‘temporal switch’ that modulates this fate decision during the maturation of the GC [108]. Considering affinity progressively increases with time spent within the GC, these findings are consistent with the observation that GC-derived plasma cells are higher in affinity than memory B cells. It has been proposed that the earlier departure and lower affinity of memory B cells compared to plasma cells may provide them with more clonal diversity and hence greater potential to protect against a wider range of pathogen variants [29, 42, 109, 110].

While differences in affinity are described for GC-derived memory B cell and plasma cell subsets, it is likely not the sole determinant of GC B-cell fate decisions [70]. Recent analysis of clones over time via use of the ‘confetti’ mouse model highlighted the diversity in affinity between GC structures, in which both low-affinity and high-affinity cells could persist for long periods of time [70]. Both low and high-affinity memory B cells were identified in the memory B-cell repertoire post-influenza infection [62], and the differentiation of memory B cells into secondary plasmablasts relied on a small number of clones with higher affinity than those that remained dormant during recall responses [24]. Further, class-switching of the constant region appears to affect B-cell selection within the GC, independent of affinity. A recent study comparing the selection of high-affinity IgG^+^ vs. high-affinity IgM^+^ B-cell clones within the same GC revealed preferential selection of IgG-switched over unswitched GC B cells [111]. Thus, how B cells differentiate into memory B cells over other fates remains to be fully elucidated. To that end, novel *in silico and in vitro* models have emerged to diversify the research toolkit, enabling new approaches to tackle questions of GC B-cell fate decisions.

## CUTTING-EDGE APPROACHES TO GC MODELLING, IN ORDER TO UNDERSTAND FATE DECISIONS AND PREDICT THE FORMATION OF IMMUNE MEMORY

### In Silico

*In silico* models of GC B-cell fate decisions can be used synergistically with traditional experimental research, both to test hypotheses that emerge from wet-lab data, as well as generate new predictions for future testing *in vivo*. A number of research groups have developed *in silico* models of the GC, with newer iterations incorporating additional molecules modulated within GC cells as those details have emerged in the literature. In 2012, an *in silico* (‘LEDA’) model was established by Meyer-Hermann *et al.* [112], modelling the process of B-cell selection and exit, based on published *in vivo* data sets such as multiphoton studies that assessed zonal cycling properties of GC B cells. This model predicts that GC B cells leave via the dark zone following a final round of cell division [112], however, the route of GC exit has yet to be formally established and some contemporary models still adhere to the ‘leave via the LZ model’ instead [113]. An updated model incorporated information about the roles of c-Myc, FOXO1 and mTOR, and tested several competing theories of T_FH_–B-cell signalling dynamics within the GC [114]. However, factors regulating memory B-cell output were not specifically tested, and thus nor were factors that may be regulating the fate decision between memory B cells and plasma cells before exiting the GC.

Newer models by other research groups have tested how memory B cell and plasma cell fate decisions may be regulated within the GC [113, 115, 116] rather than simply accounting for memory B cells in GC output [112]. A probabilistic model of the GC reaction was proposed by Rodríguez Martínez and colleagues which incorporated information about the deterministic roles of different expression levels of transcription factors, as well as stochastic interactions with other cells within the GC microenvironment [113, 115]. This model was able to replicate the temporal memory to plasma cell shift observed in [108]. Of note, while a BACH2-expressing memory B-cell precursor has been described within the GC [93], adding in BACH2 did not alter the predicted output of memory B cells [113]. Similarly, a recent computational model aimed to assess the impact and deterministic factors downstream of T_FH_–B-cell signalling on the plasma cell versus memory B-cell fate decision [116]. This latest model appeared to confirm recent work from Kurosaki and colleagues on the link between weak T-cell help and an increased potential to differentiate into memory B cells [93, 95]. In this model, transcription factor was asymmetrically distributed in daughter cells, allowing transcription factor expression levels downstream of signalling to play a role in the fate outcome of a cell. They found that an affinity-based CD40 signal was necessary to get memory B-cell output in addition to plasma cells, the temporal switch between the two subsets, and demonstrated that low-affinity memory B cell emerged before high-affinity plasma cells [116].

The success in modelling various features of a GC provides evidence for *in silico* systems to give insight into the mechanisms at the core of the GC cell fate decisions. However, it is clear that there are still numerous unknowns that must first be resolved before we are able to entirely reproduce the GC *in silico.* The incorporation of data generated in response to more complex antigens (e.g. viral infections), particularly when the resulting B-cell biology differs significantly from model antigen systems, would be valuable for ensuring applicability of models to analyse responses to different pathogens. Additionally, these models have been gaining complexity by incorporating a handful of transcription factors into the analysis. Yet, in the absence of other molecular requirements that are important for transcription factors to enact changes in gene expression (such as expression of cofactors, epigenetic regulators, chromatin conformation around target genes, etc.), understanding how the orchestration of gene expression programs that influence B-cell fate decisions is still quite limited to more simplistic deterministic assumptions. To this end, it is critical that *in silico* models are updated rigorously to reflect advances in the field that carry major implications on the representability of the model of *in vivo* processes.

### In vitro

Due to limitations involved with studying human GC activity *in vivo*, researchers have been attempting to generate a GC reaction *in vitro*, with minimal success until recently. Functional GC B cells and T_FH_ cells are notoriously difficult to generate *in vitro*, with the latter achieved using T cells possessing a TCR transgene and specific stimulatory conditions [117, 118]. In 2011, Nojima *et al.* established a culture system using 3T3 fibroblasts transfected with CD40L and BAFF, along with IL-4, that was able to generate and sustain GC-like cells (termed ‘iGB cells’) which possess some of the features of bona fide GC B cells, such as the ability to differentiate into memory-like B cells when adoptively transferred *in vivo*. By modifying levels of IL-21, the researchers in this study were able to change the developmental fate of these iGB cells from a memory B cell to a plasma cell fate [119]. However, this culture system was still a considerable way off from completely recapturing all of the features, such as 3D structure or somatic hypermutation [119]. To this end, Melnick and colleagues used the iGB setup in conjunction with hydrogel [120] to produce a 3D B-cell culture system in order to study epigenetic factors regulating the GC reaction [121]. Within this model, B cells underwent mutation and displayed a GC transcriptional program at d4, before shifting to a plasma cell program a few days later [121]. More recently, a human-derived tonsil organoid model was established in which tonsillar cells reaggregated in a transwell [122]. These organoids were able to respond to an influenza vaccine and demonstrated key features of an *in vivo* GC including zonal segregation, somatic hypermutation, affinity maturation and differentiation into plasma cells. This system should prove valuable for studying critical features of the GC under different pathogenic or therapeutic conditions using a human system [122].

The findings described above underscore the potential in new approaches for studying the GC reaction, particularly to investigate the factors that regulate fate decisions within the GC. By gaining an appreciation for these factors, we can gain a better understanding of the variability that exists between different individuals and of the factors underlying the formation of long-lived immune memory. Fully operational *in silico* and *in vitro* GC systems may eventually enable researchers to test an individual’s potential to generate immune memory to a pathogen or vaccine without infecting or immunizing that individual.

## LONG-TERM IMMUNITY TO SARS-COV-2

As COVID-19 and the roll-out of vaccines has taken centre stage around the globe, so too have numerous important questions pertaining to B-cell memory and SARS-CoV-2. Will we form neutralizing antibodies and long-lived memory B cells to this virus? If we are able to detect the presence of memory B cells, are they functional? That is, will they respond by helping to clear virus rapidly upon reinfection, protecting us from disease? Is prior exposure to other coronaviruses protective or detrimental? Finally, how long might immune protection last, and is there any way to predict this?

An improved understanding of the factors dictating humoral memory formation, along with recent advances in methodologies, has facilitated the ability to rapidly address these questions and gain detailed insight into whether we can form effective immune memory to the original strain of SARS-CoV-2 and its variants (see Table 1 e.g. of studies).

**Table 1:** Prominent technologies and methodologies that have been utilized to study B-cell responses to SARS-CoV-2

Technology/methodology	Insight	Examples of studies
Antigen probes (e.g. B-cell tetramers)	Has enabled SARS-CoV-2-specific memory B cells and their precursors in vaccinated individuals and patients with varying degrees of disease severity to be isolated and studied over time. B cells specific to different viral epitopes can be compared.	[123–130]
BCR repertoire analysis	BCR-sequencing technology has revealed superior somatic hypermutation and specificity in GC and memory B-cell clones from individuals with milder histories of COVID-19 severity and in those vaccinated against the virus compared to patients who did not recover from infection.	[124–126, 131–133]
Single-cell deep sequencing	Single-cell deep-sequencing strategies have been instrumental in advancing both phenotypic, transcriptional and BCR repertoire analyses of individual GC and memory B cells. This has improved our understanding of the different classes of B cells that respond to the virus and has led to the development of therapeutic monoclonal antibodies against SARS-CoV-2.	[129, 131–135]
Pseudo-virus assays	Have enabled researchers using biocontainment Level 2 labs to study the neutralizing potential of antibodies and B-cell clones to both SARS-CoV-1 and SARS-CoV-2.	[124, 128, 136–138]
Cryo-electron microscopy	This technology has been used to map the epitope binding sites of neutralizing antibodies to the spike protein as well as to assess cross-reactive SARS-CoV-1 antibodies.	[129, 139, 140]

### SARS-CoV-2: a novel coronavirus

There are currently four human coronaviruses that cause respiratory infections (namely, HCoV-229E, -NL63, -OC43, -HKU1), as well as three coronaviruses that have arisen as a result of zoonosis and have caused severe disease in humans, namely, SARS-CoV, MERS-CoV and SARS-CoV-2, which emerged in 2003, 2012 and 2019, respectively [141, 142]. While SARS and MERS outbreaks possessed a markedly higher fatality rate than COVID-19, 9.7% and 34%, respectively, the basic reproductive rate (R_0_, which measures transmissibility) was estimated in 2020 to be approximately 2.5 for SARS-CoV-2 but was 2.4 and 0.9 for SARS and MERS, respectively [142]. Due to this high rate of transmission and a long incubation period, SARS-CoV-2 has been able to spread around the world at a rapid rate.

### Is it possible to generate effective humoral memory against SARS-CoV-2?

**Figure 2:**
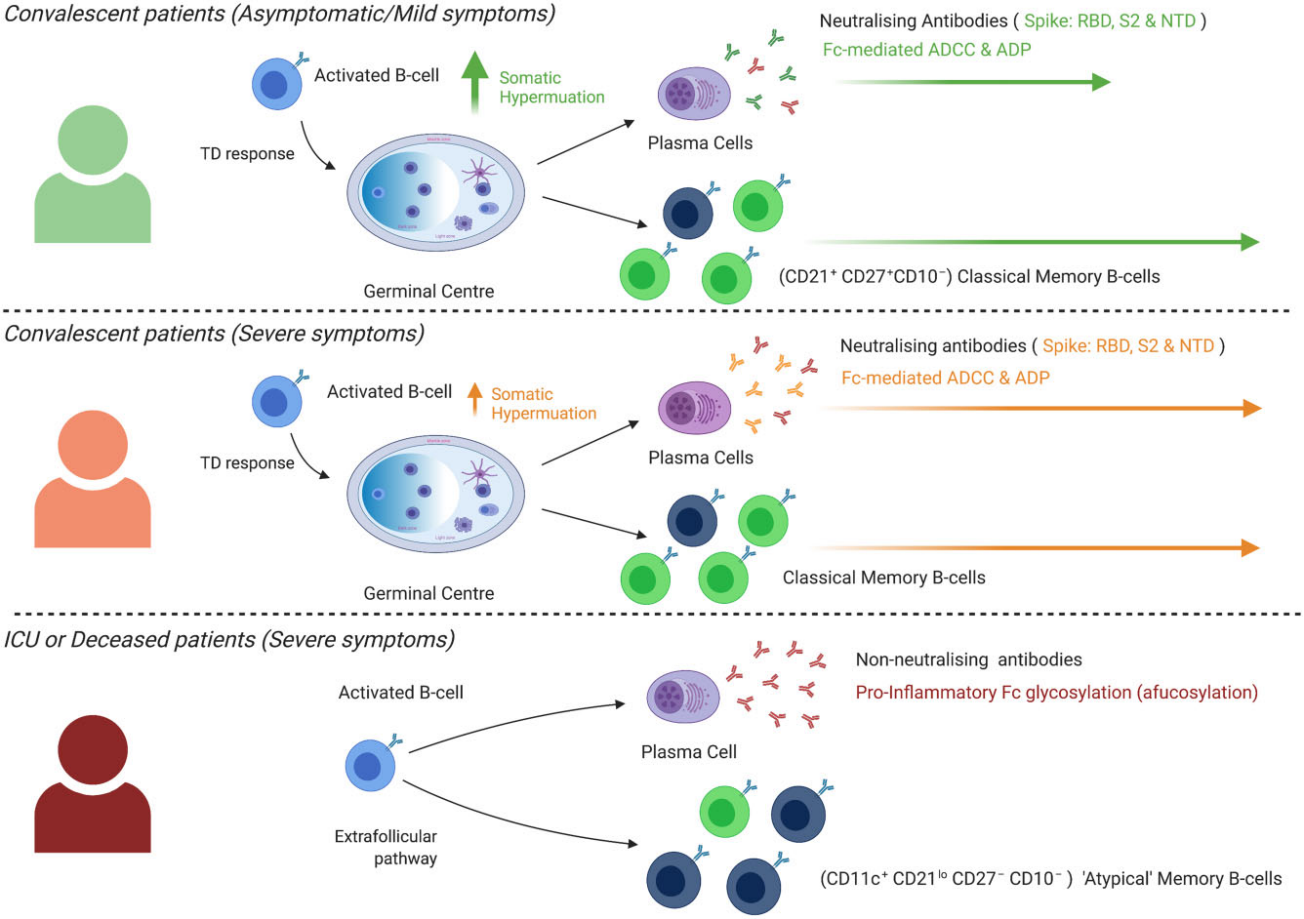
Comparing the humoral response between COVID-19 patient-groups with varying degrees of disease severity: convalescent patients who experienced mild symptoms (top section); convalescent patients who experienced severe symptoms (middle section); severely affected patients who were either in the ICU when sampled or who did not recover (bottom section).

One of the chief questions arising during this pandemic is whether an effective immune response can be mounted against SARS-CoV-2, as well as how long the immune memory generated by that response will last. Like SARS-CoV-1 and many other coronaviruses, SARS-CoV-2 gains entry to host cells via its spike (S) glycoprotein [143]. The S protein is a heterodimer composed of S1 and S2 subunits. The receptor-binding domain (RBD) is located in the S1 subunit and directly interacts with angiotensin-converting enzyme receptor 2 (ACE2) in order to achieve viral binding [144]. The spike protein, particularly the RBD, is a key target for neutralizing antibodies [145–148] and the primary immunogen of choice for vaccination efforts [149, 150] which have so far been successful at protecting individuals from COVID-19 [151, 152]. While the elicitation of neutralizing antibodies is a primary goal of modern vaccination strategies, the additional non-neutralizing effector mechanisms of antibodies also have a critical impact on disease control or progression. *N*-glycan tailoring of antibody Fc region can have either pro-inflammatory or anti-inflammatory roles by modulating Fc-mediated innate cell function [153, 154]. A number of studies have now correlated *N-*glycan tailoring with the effectiveness of the humoral response and clinical outcomes to SARS-CoV-2 [147, 155] (Table 1 and Fig. 2).

In order to track the longevity of the immune response to SARS-CoV-2, IgG antibodies specific to the virus were monitored and found to persist at robust levels for at least 148 days, 154 days and 323 days, respectively, by three different studies. In each case, IgG levels were still high when the final measurement was taken [148, 156, 157]. While the question of how long antibody levels persist will only definitively be answered with time, the duration of serum antibody levels to other members of the coronavirus family may be indicative. For example, IgG specific for SARS-CoV-1 was steady for 1–2 years before IgG levels begin to decline [158, 159]. However, serum antibody is only one component of humoral memory, and thus investigating the formation, longevity and functional capability of the memory B-cell population is key to understanding immune protection to SARS-CoV-2.

Long-term persistence of antigen-specific memory B cells in SARS-CoV-2 has been observed in convalescent patients [123, 124, 160, 161]. Multiple studies have detected RBD-specific memory B cells and T cells, suggesting that durable immunity had been established in these donors [162–164]. In another study, memory B cells and T cells were tracked for >6 months and persistent populations were observed in more than 90% of patients with varying ranges of disease severity [165]. Long-lived plasma cells have also been reported to persist in convalescent patients who experienced mild levels of disease for at least 8-months post-infection [166]. Similarly, SARS-CoV-2 mRNA vaccines induce robust memory B cells as well as neutralizing antibodies in individuals who have not previously been exposed to the virus [149]. In this latter study, it was further determined that vaccine recipients who had previously recovered from COVID-19 only required a single dose to generate peak antibody and memory B-cell levels [149]. These results confirm a functional role for memory B-cell recall responses in generating optimal levels of protective antibodies following infection or vaccination against SARS-CoV-2.

While the detection of these memory B cells is indicative of a durable humoral immune response, their presence is not a guarantee that disease has not altered their functional capability. This turns us to the next question: are there changes in the formation, phenotype or function of B-cell populations that correlate with differences in clinical outcome?

### Variations in B-cell populations correlate to variations in clinical outcome

Of central interest to researchers at the moment is the question of why some COVID-19 patients are able to mount successful immune responses and recover from infection, often without expressing any symptoms, while other, seemingly healthy individuals succumb to the disease.

As discussed in previous sections, the GC is a major site for memory formation and the generation of neutralizing antibodies. Encouragingly, there are a number of indicators that GCs form in convalescent patients, with evidence of memory B cells having undergone somatic hypermutation and selection [131, 134–136]. Using an elegant approach, Turner and colleagues were recently able to confirm the presence of RBD-specific GC B cells by performing fine-needle aspirations, at different time points, on the draining lymph nodes of participants who had been immunized with a SARS-CoV-2 mRNA vaccine [125]. These GCs were observed to persist for at least 15 weeks post-immunization, and single-cell BCR-seq analysis showed that the GC and memory B cells were somatically mutated [125]. Taken together, these studies help to demonstrate the ability of the humoral response to induce GCs and the potential to undergo affinity maturation in resolving SARS-CoV-2 infection or to immunization.

In contrast, several other studies over the last year have made markedly different observations when studying samples from individuals that were unable to recover from COVID-19. Strikingly, Kaneko *et al.* revealed an absence of GC B cells or T_FH_ cells in the lymphoid tissue of recently deceased COVID-19 patients [167]. GC B cells and T_FH_ cells were also undetectable within draining lymph nodes isolated from deceased patients [168], and antibody levels significantly declined in the days before death in a separate cohort [169]. While it is certainly likely that the inability to mount a GC response to SARS-CoV-2 may have contributed to the clinical outcome of these patients, this still remains to be determined. Fortunately, it seems these cohorts are not representative of the majority of patients who contract the disease and recover, who seemingly generate GCs given that long-lived plasma cells, neutralizing antibody, and a persisting memory B cell population have all been detected [125, 135, 136, 165, 170].

If GCs are dysregulated or absent in severe patients, as reported by Kaneko *et al.*, then these memory B cells formed would presumably be GC-independent. This theory would align with a study by Woodruff *et al.* who characterized B-cell subsets in patients with severe disease and described a prominent CD11c^+^CD21^lo^ phenotype, a similar phenotype to that of extrafollicular-derived B cells in systemic lupus erythematosus patients [171], as well as to the phenotype described for atypical memory B cells in different chronic or recurrent infectious settings (reviewed in Ref. [32]). Similarly, CD21^lo^CD27^−^CD10^−^ memory B cells were expanded in patients severely affected by COVID-19, although antigen-specificity was not determined [172, 173]. An expansion of unmutated IgG^+^ memory B cells has been observed in patients with severe disease [131], and the lack of IgM^+^ memory B cells in hospitalized COVID-19 patients was also correlated to poor clinical outcome [174]. These studies are supportive of a model in which B-cell pathways are dysregulated in severe patients.

Interestingly, a number of findings seem to indicate that convalescent patients that had been severely affected by COVID-19 are capable of producing higher levels of RBD-specific antibodies with neutralizing capacity and memory B cells than mild or asymptomatic SARS-CoV-2 infection [175–177]. In these instances, it seems that disease severity may not be easily explained by the reduction or lack of a humoral response targeted against the virus. Serum antibody titres decline at a much faster rate (∼4 months), in convalescent patients with a mild history of infection than in those who experienced more severe symptoms (who can maintain robust titres for at least a year post-infection) [157, 178]. The decline of serum antibody led researchers to isolate memory B cells from the PBMCs of convalescent patients, including several whose IgG levels had declined past the point of detection [179]. Memory B cells capable of generating neutralizing antibodies were found to persist in the blood of all the convalescent patients in the study, regardless of serum antibody levels [179]. These findings highlight the limitations involved with using serum antibodies as the sole measure of humoral immunity to a particular pathogen. At the same time, the capacity of functional memory B-cell responses offers promise for our potential to generate long-term immunity in response to both mild and severe COVID-19 infection.

While there are still a number of significant gaps in our knowledge about the changes in B-cell responses that occur in different cohorts defined by patient severity, together this data suggests that clinical outcome is correlated to the ability of the patient to generate an effective GC-dependent humoral immune response (see Fig. 2).

### Does cross-reactivity from other coronaviruses occur and is this protective?

One prominent question that has arisen while studying the B-cell response to SARS-CoV-2 infection, or the vaccines recently developed to protect against this virus, is whether previous exposure to other coronaviruses would be protective or detrimental to the host response. A major way that cross-reactive humoral responses could be detrimental is via ‘Original Antigenic Sin’. That is, if activation of a cross-reactive memory B cell produced against a distinct, but antigenically similar virus or viral strain impedes the generation of an effective primary response to the pathogen at hand [180] and/or leads to antibody-dependent enhancement during infection or vaccination [153]. While evidence of any substantial role of the latter in this pandemic is currently lacking, a recent study determined that convalescent subjects that had severe symptoms contained a significantly expanded population of antibody-secreting cells targeted towards seasonal coronavirus spike proteins, compared to those with mild disease [134]. Further, Sen and colleagues have detected antibodies to an epitope (Ep9) of the SARS-CoV-2 nucleocapsid that is bioinformatically predicted to be similar to that of the neuraminidase protein of the H3N2 influenza virus [181]. Ep9-specific antibodies produced in response to SARS-CoV-2 are correlated with increased disease severity [182]. However, it remains to be determined whether the reactivation of Ep9 clones or cross-reactive clones specific for endemic coronaviruses directly impairs the formation of effective responses to infection or vaccination to SARS-CoV-2.

In fact, various studies have identified cross-reactive B-cell clones and antibody in productive responses to SARS-CoV-2 infection or vaccination [125, 127, 132]. Considering the conserved mechanism for cell entry and hence conserved neutralizing antibody target, researchers had hypothesized that previous exposure to other coronaviruses might be protective for patients. Cross-reactive responses had previously been observed with SARS-CoV-1. In 2005, Chan *et al.* reported that infection with SARS-CoV-1 resulted in the increases of sera antibody against other coronaviruses 229E, NL63 and OC43 [183]. Notably, Zhu *et al.* tested serum collected from 20 convalescent patients infected with the 2003 SARS-CoV-1 virus and observed cross-reactive neutralizing antibodies against key components of the SARS-CoV-2 spike domain [184]. These findings are in concordance with the recent study on SARS-CoV-2 vaccines, showing that B-cell clones cross-reactive to OC43 and HKU1 were detected in sampled GCs post-immunization [125]. In this study, comparative binding analysis of monoclonal antibodies revealed that while the majority of clones were specific for SARS-CoV-2, a select number were found to be cross-reactive to the more common coronaviruses and possessed significantly more somatic mutations [125]. The authors suggested that these latter clones were recalled memory B cells, formed during a previous infection [125]. Together, these results leave open the possibility that prior exposure to other coronaviruses may offer protection by providing an avenue for the rapid production of S-specific neutralizing antibodies by cross-reactive memory B cells [125]. Thus, the restimulation of cross-reactive clones does not appear to be a prominent obstacle in the generation of long-term, high-affinity memory to SARS-CoV-2.

## CONCLUSION

There have been several landmark studies that have transformed our understanding of the regulatory dynamics that underlie the GC and the factors regulating the fate of its constituent cells. Significant breakthroughs in the field of GC and memory B-cell biology over the course of the last few decades, along with the advent of novel tools with which to study these cells, have put us in better stead to understand the humoral response to newly emerging pathogens than ever before. There do still exist, however, a number of critical unknowns which preclude us from confidently predicting or administering long-term immunity in patients. The deciding factors which determine whether a B cell departs the GC as a plasma cell or as a memory B cell remain so far unresolved. As a result of this and several other unknowns, computational models of the GC reaction have been unable to recapture all of the features of these structures *in silico*, but with new knowledge have the potential to be used in the future to predict the dynamics of memory B-cell formation. Attempts to model the GC using *in vitro* systems have had success with 3D organoid cultures, demonstrating their potential utility to predict the ability of vaccines to elicit clonal selection and memory formation. Notwithstanding these advances, more work is required before immune memory can be tested and predicted using these systems.

Despite the rollout of vaccines designed to elicit lasting immunity to COVID-19, there still remain many questions surrounding the mechanisms by which the humoral immune system generates lasting humoral memory to SARS-CoV-2. While there appears to be a disruption either in the formation or persistence of GCs in deceased patients, we do not yet know precisely why convalescent patient cohorts are more capable of generating robust and healthy GC responses and thereby long-term immunity. While SARS-CoV-2-specific memory B cells have been detected in patients following COVID-19 infection, the protective quality of these cells largely still remains to be tested via secondary recall. As of yet, there is still no unique biomarker that can be used to predict the longevity or efficaciousness of a memory B cell. However, as we gain more understanding about the factors that underpin the formation and fate of long-term immunity, certain molecules have begun to emerge, such as T-bet, which have the potential of being used to predict the success and duration of the memory B cells produced by the infection or vaccine. Additionally, it remains to be determined how memory B cells with differing phenotypes, such as ‘atypical’, extrafollicular or tissue-resident memory B cells, can affect the course of the disease. By reviewing recent breakthroughs in our understanding of how lasting humoral memory is generated and by utilizing novel analytical tools for studying the B cell response to SARS-CoV-2, we can address some of these unknowns and improve our chances of overcoming this pandemic as well as the next.
